# Osteogenesis Imperfecta and Child Abuse From a Forensic Point of View

**DOI:** 10.7759/cureus.12790

**Published:** 2021-01-19

**Authors:** Abdulraheem Altalib, Abdulrahman Althomali, Abdulrhman Alshahrani, Abdullah Alfrayyan, Musaad S Aljughaiman

**Affiliations:** 1 Orthopedics, King Fahad Hospital of the University, Khobar, SAU

**Keywords:** osteogenesis imperfecta, child abuse, fragility

## Abstract

Osteogenesis imperfecta (OI) also called brittle bone disease is a rare genetic disorder that results from a defect in type 1 collagen, which is a main structural protein involved in the structure of bones, tendons, ligaments, the dentin layer of teeth, and the sclera of the eye. The defect in this gene is known to be a predisposing factor to fractures. The deficiency in type 1 collagen can be either qualitative or quantitative. Due to this deficiency, the bones become so fragile and can break easily with minimal trauma, which can be coined as “imperfect bone formation.” It also leads to bruises due to the extravasation of blood in the connective tissue of the skin. Sometimes, fractures may result from the routine handling of parents. It can be misleading since fractures are considered to be the second most common sign of child abuse according to the literature. One of the main duties in forensic medicine is examining live victims, which plays a crucial role in confirming a clinical diagnosis. In this paper, a review of the literature was conducted and a summary of reported cases of osteogenesis imperfecta, which were initially diagnosed as child abuse, is presented.

The aim of this study was to review the literature for the prevalence of misdiagnosed cases of osteogenesis imperfecta as child abuse, analyzing various types of presentations in osteogenesis imperfecta that might lead a physician to a wrong diagnosis of child abuse and to clarify common findings and fracture sites seen among patients with osteogenesis imperfecta. The literature review was conducted for both conditions, osteogenesis imperfecta and child abuse, and an evaluation and analysis of case reports and case series regarding osteogenesis imperfecta cases misdiagnosed as child abuse utilizing the PubMed search engine.

Unexplained fractures in children validate the consideration of osteogenesis imperfecta and child physical abuse. A thorough and careful evaluation is recommended as soon as possible because a delay can result in psychological consequences for both the child and the family.

## Introduction and background

Osteogenesis imperfecta (OI), also known as brittle bone disease, was first described by Malebranche in 1678 but soon got its name from Vrolik, a Dutch anatomist. OI is a group of disorders that is thought to be caused by a defect in collagen type 1. OI is an uncommon congenital disease, yet it is considered the most common inherited disorder, which primarily involves bones. The main features of OI are bone fragility and skeletal deformities. Other manifestations include dental abnormalities, bluish discoloration of the sclera, laxity of the joints, and deafness, which is mainly due to a defect among the three small bones of the middle ear [1]. Patients presenting with fractures at a young age yield a high level of suspicion. There have been lots of controversies regarding the diagnosis of OI since it is quite rare, and there have been cases that were misdiagnosed as child abuse. OI can be misinterpreted as a form of physical abuse among children. Differentiating OI from child abuse, particularly in the form of physical abuse as manifested by non-accidental fractures, can be a difficult task for most physicians. These two conditions may overlap, as they are highly relevant differential diagnoses when a child presents with an unexplained fracture. A definitive diagnosis must be established; otherwise, confusion and misdiagnosis can hinder management and lead to devastating emotional damage to the family [2-3]. Aside from being accused of juvenile maltreatment and losing custody over their children, involved families also suffer from the scrutinizing judgment of society, which can lead to serious and aggravating effects, especially to the parents [4]. With that being said, healthcare providers, especially doctors, should be competent in diagnosing child abuse through a comprehensive examination, use of necessary diagnostic tools, in-depth history-taking, and, most importantly, having broad knowledge of the genetic differentials of child abuse [5-6]. This paper will tackle the controversies of OI and child abuse: correlation, misdiagnosis, and false accusations. We will also demonstrate case reports regarding these issues.

Materials and methods

**Figure 1:**
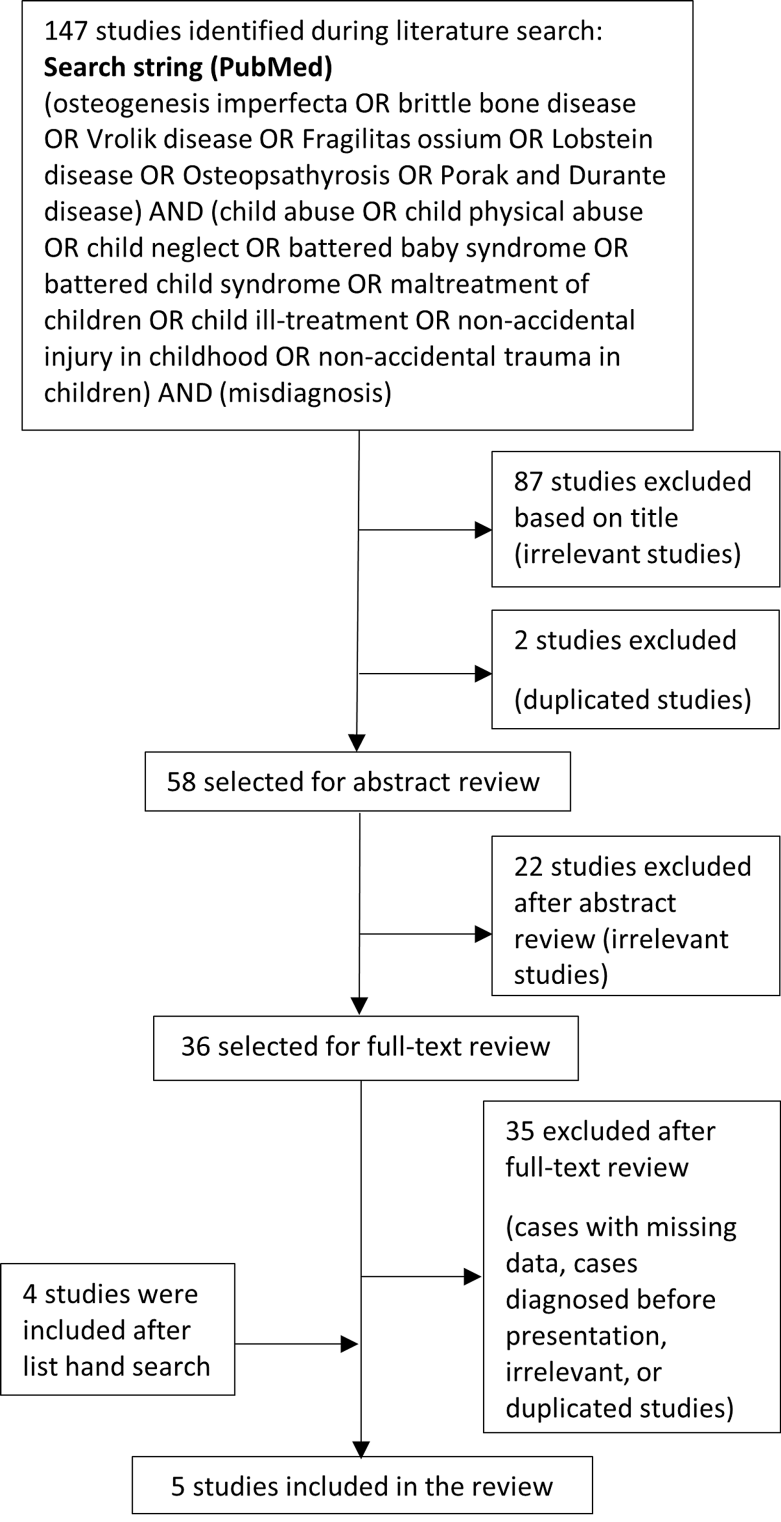
This flow diagram presents the review process used in this study

A literature review was conducted for both conditions: osteogenesis imperfecta and child abuse and an evaluation and analysis of case reports and case series regarding osteogenesis imperfecta cases misdiagnosed as child abuse utilizing the PubMed search engine. Results were sorted by Best Match (Figure 1).

## Review

Definition and clinical manifestations of osteogenesis imperfecta and child abuse

Child Abuse

In 2010, the Centers for Disease Control and Prevention (CDC) defined child abuse as any act, intentional or not, that results in harm, the potential for harm, or the threat of harm to a child. The failure to provide for a child’s needs or to protect a child from harm or potential harm is also child maltreatment. Child abuse could be carried out by a parent, a caregiver, or an authorized custodian of the child. Fracture is considered one of the most common signs of child abuse, which occurs in over 50% of maltreated children [7]. Other signs include retinal hemorrhage [8], facial burns, lacerations of the lips and lingual frenulum, and bite marks on the face and neck areas [9]. It occurs in over 50% percent of abused children [10]. Multiple fractures in areas such as the hips, humerus, femur, and skull, are evident signs of physical abuse. The most usual form is the linear fracture [11]. In 2019, the World Health Organization (WHO) categorized child abuse or maltreatment into four types: physical abuse, sexual abuse, emotional abuse, and neglect. Each one is different, yet harmful to the children and families involved and their communities.

In order to determine the attitudes of healthcare workers in reporting child abuse, one study was done in Saudi Arabia. A total of 327 professionals participated in this case study, with doctors/nurses accounting for 24% of the total study, 20% were therapists/psychiatrists, a significant 24% share from social workers, 17% were educators, and small percentages were law enforcement professionals and medical examiners, 9% and 5%, respectively. Attitude scores varied significantly based on the correspondents' gender, specialty, and training. Overall, it was seen that women, healthcare workers, and those individuals who were trained in more than five courses related to child abuse showed more concern in the under-reporting of juvenile abuse. On the other hand, men, medical examiners, law enforcement staff, and undertrained professionals showed a tendency to not fully report alleged cases of child abuse [12].

A retrospective study about the incidence and types of emotional abuse among children was conducted in Saudi Arabia. The correspondents ranging from 12 to 18 years of age were acquired from three different malls in Jeddah, a huge city in Saudi Arabia. The said test concluded that 90% of participants experienced emotional abuse through rejection, whereas 61.7% of the total study reported an incidence of ignoring or terrorizing forms of abuse. Furthermore, it shows that the long-term illness of the children's parents had positive implications with the terrorizing type of emotional abuse while the mother-child relationship posed a negative relation with emotional abuse [13].

In 2018, research was conducted in various secondary high schools in Saudi Arabia with a goal to investigate the percentage of sexual abuse in the said country. The participants' mean age was 16.8 years with 50.8% boys as part of the entire study. Through this national survey, which was based on the International Society for Prevention of Child Abuse Screening Tool Children's version, they found that 16% of the total participants experienced child abuse in their lives. Those children who lived with their step-parents had a higher risk of experiencing sexual abuse as compared to those who dwelled with their biological parents [14].

Furthermore, research among 300 primary health care physicians in Abha, Saudi Arabia, was conducted to gauge their knowledge of child abuse and behaviors in reporting such cases. All participants (65% males, 69% married, and 73% with at least one child) were given self-administered questionnaires during their time between patients' check-ups. As a result, 96.3% of them had good knowledge of the types of child abuse. Additionally, 97.3% showed an understanding of child abuse patterns. However, more than half of them revealed underreporting of child abuse [4], Another retrospective study about the types of family profiles of children who experienced abuse and neglect was performed. The information was obtained from the Child Protection Center in King Abdul-Aziz Medical City, Riyadh, Saudi Arabia, dated from July 2009 to December 2013. Four main criteria were used in this research such as the demographics of the victim, family profile, parental information, and information on the perpetrator and forms of abuse. Among 220 cases of child abuse and neglect, physical abuse topped the list with 42%, an alarming rate of 39% due to neglect, 14% from sexual abuse, and 4% because of emotional abuse, which was the least form of abuse. In terms of sexual abuse, it was found that boys were more likely to experience it than girls. Moreover, children staying in larger family households were 1.5 times more likely to feel neglected than those who lived in smaller households. Those who had unemployed fathers had a significantly greater risk (2.8 times) of experiencing physical abuse. Living with single or stepparents was also seen as a risk factor (4.8 times) that could lead to physical abuse [15].

Osteogenesis Imperfecta

It is a group of heterogeneous inherited connective tissue disorders that cause a variety of skeletal and extra-skeletal abnormalities. Skeletal manifestations include fragile bones and low bone mass [16]. Extra-skeletal manifestations include blue sclera, Wormian bones, hearing problems, dentinogenesis imperfecta, and loose ligaments. The presence of bluish sclera and Wormian bones increases the suspicion of OI. The severity level ranges from mild to lethal. There are four main types of OI (types I-IV), and they are based on clinical, radiological, as well as genetic data. Also, there are other additional types of OI that have existed and have been identified in some patients (types V-VII) [17].

Osteogenesis imperfecta is classified into seven types: Type I is the most common type among other types of OI. The patient typically presents with a mild form of the disease in the absence of major bone deformities. This type is subdivided into type A, which is the absence of dentinogenesis imperfecta, and type B in which the patient has dentinogenesis imperfecta. Sclerae turning blue and bone fractures usually occur before puberty. The patient typically can have a mild form of scoliosis as a result of vertebral fractures. There is a 50% chance of the occurrence of hearing loss among families. The hypermobility of joints and thinning of the skin are present. They have a normal level of intelligence and life expectancy is not affected [18]. Regarding Type II, in the perinatal period, it is considered to be lethal as a result of respiratory failure from rib fractures. The main feature of this type is a frog-like position. Common signs are bluish sclera and multiple fractures with deformity involving the extremities, and the patients do not usually live after one year [18]. In Type III, they have multiple fractures that cause short stature and deformities involving the spine and limbs. They have a problem with respiration, which is the leading cause of death. There is normal sclerae, an asymmetric head, and a triangularly shaped face. A posterior inclination of the maxilla is present in almost 80% of the cases, and this group of patients lives into adulthood [18]. While in Type IV, the severity ranges between types I and III. The deformities are usually ranging from mild to moderate. Sclerae are gray in color with dentinogenesis imperfecta [18]. In Type V, sclerae are normal, and there is no evidence of dentinogenesis imperfecta. There is a limitation of movement in the forearm, which may lead to radial head dislocation [18]. Type VI is based on histological findings. There is an increased amount of osteoid, which is above normal [18]. The last is Type VII, which is considered a moderate form of deformity. In the infant period, they present with coxa vara. Rhizomelia is a common feature in this type [18].

Diagnosing osteogenesis imperfecta is based on a thorough history, physical examination, radiological findings, and genetic testing that may be required to confirm the diagnosis. The clinical course is helpful in narrowing the differential diagnosis. The hallmark finding in osteogenesis imperfecta is mild trauma leading to fractures, bowing of long bones, and growth restriction [19]. The clinical features of osteogenesis imperfecta depend on the age of the patient and the severity of the disease. The skeletal features of osteogenesis imperfecta vary among different types but there can be flattening of the midface, macrocephaly, dentinogenesis imperfecta, and chest wall deformities [20]. Osteogenesis imperfecta extends beyond skeletal features showing non-skeletal manifestations such as bluish discoloration of the sclera, hearing abnormalities, impaired pulmonary function, and regurgitation of the cardiac valves. Also, a positive family history of dentinogenesis imperfecta, bone fragility, or hearing impairment can help in diagnosing a patient with osteogenesis imperfecta. Radiological findings are generalized osteopenia, Wormian bones, and deformity of the chest wall [21]. The role of genetic testing in patients with a high suspicion of osteogenesis imperfecta is to rule out firstly COL1 genes. Thus, this will reduce the economic burden and will detect most cases of osteogenesis imperfecta. Molecular testing is helpful in knowing the recurrence rate in offspring and the prognosis of the disease. Establishing a diagnosis of osteogenesis imperfecta can sometimes be straightforward because of typical findings and specific pathognomonic, but in cases where fractures are found in isolation, diagnosis can be difficult and requires a multidisciplinary approach and forensic evaluation. A diagnostic approach for such patients may be difficult in some cases and misdiagnosing. Osteogenesis imperfecta for child abuse is not uncommon and can be a disaster for families [4].

Results

After a thorough literature review (Table 1), two case series that include 27 cases written by C. Paterson in 1989 and in 2006 [22-23] were identified. Furthermore, three cases of osteogenic imperfecta misdiagnosed initially as child abuse were reported by K. Ojima (1994) [1], H. Minnis (1995) [24], and D’Eufemia (2012) [3].

**Table 1:** Case reports and case series of osteogenic imperfecta misdiagnosed with child abuse Acronyms: R’ = Reference, R = Right, L = Left, += Positive, 0 = Negative, - = Not mentioned, F = Female

Case reports and case series of osteogenic imperfecta misdiagnosed with child abuse											
#	Age at first presentation	Gender	Probable type	Family history	Scleral color	Wormian bones	Dentinogenesis imperfecta	Fractures	Consequences	Follow up	R’
1	10 months	-	IA	0	Blue	-	0	L.Ulna	Case conferences. At risk registered three years. Two admissions to hospital for observation	Continues to fracture	22
2	3 weeks	-	IA	0	Blue	>20	0	Ribs, R. Femur	Case conferences. Place of safety order	Fractured in hospital and continuous to fracture	22
3	21 Months	-	IA	0	Blue	>10	0	R.Tibia, R. humerus	Case conferences. At risk registered for several years	Further regular fractures	22
4	4 Month	-	IB	+	Blue	0	+	L.Femur	Case conferences. Care proceedings prosecution proposed but dropped	No further fractures	22
5	6 Months	-	IB	0	Blue	>20	+	R.Femur, L.tibia	Case conferences. Detention in hospital	Further fractures (one in hospital)	22
6	4 Weeks	-	III	0	Blue	-	+	R.Femur	Case conferences. Detention in hospital	Further fractures ( in hospital), subsequent fractures.	22
7	3 Weeks	-	IVA	+	Pale Blue	0	0	Ribs, clavicle, acromion, L&R Radius	Case conferences. Interim care order. Later discharge at local authority’s request	One further fracture	22
8	10 Months	-	IVA	0	White	>20	0	R.humerus, R.Femur	Case conferences. Care proceedings prosecution proposed but dropped. Care order made adoption proposed	No fractures for 18 months, then further fractures in foster care.	22
9	6 Weeks	-	IVA	+	Pale blue (later become normal)	4	0	L.humerus, L.radius, Skull, R.Femur, L.Radius and ulna	Case conferences. Parents prosecuted but acquitted. Care proceedings twice taken into care	Further infrequent fractures until age of twelve	22
10	33 Months	-	IVA	+	White	-	0	L.tibia,	Case conferences.	20 further fractures over 8 years	22
11	15 months	-	IVA	+	White	1	0	L.femur and tibia	Case conferences. Police investigation. No prosecution. At risk register.	No further fractures	22
12	5 months	-	IVA	0	White	6	0	R.femur, Rib fractures	Case conferences. Detention in hospital.	Further fracture in hospital and many subsequent fractures	22
13	11 months	-	IVA	+	White	7	0	R,L.humerus	Case conferences. Care proceedings. No order made.	No further fractures	22
14	10 months	-	IVB	0	Pale blue (later become normal)	6	+	R.femur	Case conferences. At risk register. Police inquiries.	Further regular fractures including skull fracture	22
15	7 weeks	-	IVB	0	Pale blue (later become normal)	>10	+	R.femur	Case conferences. Police inquiries.	Further fracture in hospital and many subsequent fractures.	22
16	4 Weeks	-	IA	+	Blue/gray	2	-	R.femur L.clavicle	Foster care for 3 months. Six fractures in foster and hospital care. Returned to parents after care proceeding.	4 fractures in 5 years. Otherwise good progress.	23
17	10 Months	-	IB	+	Blue	6	-	R.Femur, R.tibia	Formal finding of abuse but returned to mother. Finding reversed on appeal.	No fractures in 4 years. Sclerae remained blue. Dentinogenesis imperfecta became evident.	23
18	3 Months	-	IB	+	Blue	0	-	14 rib fractures, R.Radius and humerus. L.tibia and fibula	Care proceeding, returned to parents.	Continuous fracture over next 5 years	23
19	At birth	-	IA	0	Pale blue	0	-	Clavicle. L.femur	Initially fostered with grandparents. Supervision order refused by court. Returned home.	No fracture over the next 4 years. Sclera remain abnormal	23
20	15 Months	-	IVA	0	Pale blue	>20	-	R.Tibia and fibula. L.femur and tibia	Care proceedings. Remained with parents.	Continued to have infrequent fractures to age 11years.	23
21	6 months	-	IA	0	blue	>20	-	R.humerus, L.tibia	At risk register. No care proceedings.	No fracture over the next 4 years. Sclera remain blue.	23
22	5 months	-	III	0	blue	>20	-	Five ribs fractures	Care proceedings. Foster care from age 12 months to age 17 months.	More than 30 fractures in next 5 years. Diagnosis of OI type III confirmed.	23
23	3 weeks	-	IA	0	blue	0	-	R.femur, L.humerus	Care proceedings. Foster care. Gradual return to parents at 2 years.	No fracture over the next 2 years. Sclera remain abnormal.	23
24	9 months	-	IVA	+	Pale gray	6	-	R,L femur	Care proceedings. Case conference: register at risk. Prolonged stay at hospital.	At least one additional fracture.	23
25	18 months	-	IVA	+	white	4	-	L.femur	remained with parents	No fracture over the next 3 years.	23
26	18 months	-	IVA	+	white	2	-	R.humerus,L.tibia	One prolonged stay in hospital. remained with parents	Supracondylar fracture L. humerus at age 4 years.	23
27	2 months	-	IVA	0	white	>20	-	L.femur, L.clavicle	Care proceedings: fostered with relatives.	Fractures of both femurs aged 8 months. Returned to parents. No fracture over the next 3 years.	23
28	3 weeks	F	III	0	Blue	-	-	Both femurs and ribs fractures.	Emergency protection order. Foster care.	Additional fracture in foster care.	24
29	20 months	F	-	0	Blue	2	-	Skull. Supracondylar humerus. L. radius and ulna. R. tibia.	Diagnosis of OI delayed. Case was reported to the authority as suspected case of child abuse and serious investigation took place.	Responded well treatment.	3
30	35 months	F	III	-	White	+	+	Arms.	Found in cardiac arrest condition. Resuscitation failed. Autopsy was taken because of multiple untreated fractures.	-	1

In this reviewed article, 30 cases, shown in Table 1, concludes that the mean age at presentation was 37 weeks (SD±37). The earliest presentation among the recorded cases was immediately after birth. On the other hand, the latest first presentation was at 35 months (Table 2). 

**Table 2:** Age at presentation

Age at presentation	
Age (weeks)	
140	Maximum
At Birth	Minimum
37 (±37)	Mean (SD)

The commonest type was IVA, which occurred in 12 cases (40%), followed by IA, IB, III, and IVB (7, 4, 4, 2, and 1, respectively). Only 40% of cases have a positive family history. Since bluish scleral discoloration and Wormian bones are characteristics of osteogenic imperfecta, 21 cases were found to have blue sclera and Wormian bones formation at presentation, whereas only six cases were found to have dentinogenesis imperfecta (Tables 3-4).

**Table 3:** Osteogenic Imperfecta types and number of cases recorded Acronyms: OI = Osteogenic Imperfecta

Osteogenic imperfecta types and number of cases recorded	
Number of Cases	OI Type
7	IA
4	IB
4	III
12	IVA
2	IVB
1	Not Verified

**Table 4:** Presence of the characteristics of osteogenic imperfecta in the recorded cases

Presence of the characteristics of osteogenic imperfecta in the recorded cases						
Dentinogenesis Imperfecta		Wormian Bone	Scleral Discoloration	Family History		
6		21	21	12	Positive	
10		5	9	17	Negative	
14		4	0	1	Not Verified	

The most fractured bone in osteogenic imperfecta recorded in this review was the femur. It is found in 19 patients or 64% of the cases. This is followed by the tibia, humerus, ribs, and radius (33%, 27%, 20%, and 17%, respectively) (Table 5).

**Table 5:** The incidence of fractures that occurred in each bone recorded in the cases

The incidence of fractures that occurred in each bone recorded in the cases	
# Of Cases (Percentage)	Fracture Site
19 (64%)	Femur
10 (33%)	Tibia
8 (27%)	Humerus
6 (20%)	Ribs
5 (17%)	Radius
4 (13%)	Clavicle
3 (10%)	Ulna
2 (6%)	Skull
2 (6%)	Fibula
1 (3%)	Acromion

Discussion

Osteogenesis imperfecta raises suspicion for two serious issues from a medicolegal point of view. The first being whether this child presenting with an unexplained fracture is a victim of battered child syndrome or not since fractures in various parts of the skeletal system are one of the most frequent manifestations of this syndrome after skin injuries. Differentiating these two conditions, although sometimes challenging, is very crucial, and misdiagnosing one for another can have a psychological, social, and medical impact on the patients and families and further judicial complications [25-26]. 

The second issue is predicting the effect of the fracture or traumatic lesion linked to a diseased patient. Interpreting a traumatic lesion should include different aspects of an injury; first, the intensity of trauma: a low-intensity trauma associated with fracture can lead toward osteogenesis imperfecta, taking into consideration other soft tissue or skin lesions around the site of fractures. Second, healing time: although in osteogenesis imperfecta, the healing time is not significantly different from normal people, prolonged immobilization following a fracture is of clinical necessity since the complication rate is higher than in normal people. And finally, complication rate: patients with osteogenesis imperfecta have higher rates of developing joint stiffness and early arthritic changes, which results in a significantly higher incidence of pain [25].

Since osteogenesis imperfecta is known to alter the bone quality, it can be very difficult from a medico-legal perspective to assess traumatic lesions found in this disease entity. For forensic evaluation to be highly precise, there must be a comprehensive medical evaluation established, a history regarding pre-traumatic status should be conducted, and a head-to-toe examination should be done with a thorough evaluation for any associated soft tissue injuries or marks, which has medicolegal importance regarding the kinetic energy associated with traumatic events. Skeletal radiographs are of important value because old fractures are usually missed during physical examination. A full description during the healing process and if any anomalies or complications were noticed should be documented. Whenever suspicion is raised, it is of legal importance to confirm or exclude osteogenesis imperfecta by biochemical testing [25,27].

## Conclusions

Osteogenesis imperfecta displays a challenge in terms of forensic evaluation, especially in cases where fractures are the only sign found in these patients. Differentiating osteogenesis imperfecta and child abuse is difficult from a medicolegal perspective, and it is very serious, as it can determine the fate of a child and have a devastating effect on the parents. Most published papers regarding osteogenesis imperfecta include child abuse as one of the differential diagnoses, and this shows how significant child abuse is in regard to misdiagnosing osteogenesis imperfecta.
